# Sensory-substitution based sound perception using a spinal computer–brain interface

**DOI:** 10.1038/s41598-024-75779-2

**Published:** 2024-10-22

**Authors:** Gabriella Miklós, László Halász, Maximilian Hasslberger, Emilia Toth, Ljubomir Manola, Saman Hagh Gooie, Gijs van Elswijk, Bálint Várkuti, Loránd Erőss

**Affiliations:** 1 Institute of Neurosurgery and Neurointervention, Faculty of Medicine, Semmelweis University, Budapest, Hungary; 2 János Szentágothai Doctoral School of Neurosciences, Semmelweis University, Budapest, Hungary; 3 CereGate GmbH, München, Germany; 4 Albert Szent-Györgyi Medical School, Doctoral School of Clinical Medicine, Clinical and Experimental Research for Reconstructive and Organ-Sparing Surgery, University of Szeged, Szeged, Hungary

**Keywords:** Sensory substitution, Sound, Computer–brain interface, Brain–computer interface, Sensory processing, Somatosensory system, Rehabilitation

## Abstract

Sensory substitution offers a promising approach to restore lost sensory functions. Here we show that spinal cord stimulation (SCS), typically used for chronic pain management, can potentially serve as a novel auditory sensory substitution device. We recruited 13 patients undergoing SCS implantation and translated everyday sound samples into personalized SCS patterns during their trial phase. In a sound identification task—where chance-level performance was 33.3%—participants ($n=8$) achieved a mean accuracy of 72.8% using only SCS input. We observed a weak positive correlation between stimulation bitrate and identification accuracy. A follow-up discrimination task ($n=5$) confirmed that reduced bitrates significantly impaired participants’ ability to distinguish between consecutive SCS patterns, indicating effective processing of additional information at higher bitrates. These findings demonstrate the feasibility of using existing SCS technology to create a novel neural interface for a sound prosthesis. Our results pave the way for future research to enhance stimulation fidelity, assess long-term training effects, and explore integration with other auditory aids for comprehensive hearing rehabilitation.

## Introduction

Millions of people worldwide experience moderate to complete hearing loss, significantly impacting their ability to communicate and connect with the world around them. Due to the aging population, hearing loss prevalence is expected to rise further^1^. It is estimated that by 2050 over 700 million people—or 1 in every 10 people—will have disabling hearing loss^2^. For a subgroup of those people, a hearing aid will not provide sufficient benefit. This is particularly the case when, for example, hair cells and inner ear structures are damaged or missing^3^; in such situations, other solutions are required. While cochlear implants (CI), which have gained widespread global use, can offer a remarkable solution in some cases^4,5^, these may not be suitable or available for all patients^6^. Furthermore, a recent analysis by Carlson et al.^5^ indicated that up to 16% of adult cochlear implant (CI) recipients had poor long-term outcomes, defined as achieving less than 30% in word or sentence recognition scores at the 12-month mark. Another study reported that 35% of experienced recipients were unable to use the telephone^7^.

Auditory Brainstem Implants (ABI) can sometimes be considered as an alternative when CI is not a viable option^8,9^. Although globally more than one thousand individuals have received ABI by now^9^, this is far fewer than CI, with over fifteen thousand recipients per year in the US alone^10^. Given the high prevalence of disabling hearing loss, its significant societal impact, and because not all patients can benefit from existing treatments, it is clearly important to develop and refine a diverse range of treatment options to meet the needs of all patients.

Sensory substitution, which involves converting information from one sensory modality into stimuli that can be processed and integrated through another sense, offers a promising approach to convey sound through means other than the auditory sense. Sensory substitution devices have emerged as a potential solution to restore sensory functions that are lost due to injury or disease^11,12^. Research in this field has demonstrated successes in utilizing auditory^13–15^ and tactile stimulation^16–18^ to substitute visual information. Additionally, sensory substitution could help alleviate vestibular dysfunction and other balance disorders^19,20^. Therefore, sensory substitution may also be helpful for people with hearing impairments.

Sound-to-touch conversion is a promising substitution method for enhancing sound perception and communication abilities^21^. Spatiotemporal vibrotactile patterns applied to the skin of the dorsal body regions can effectively convey sound information^22^. Individuals using a wearable vibrotactile device have been shown to successfully identify sounds through associated vibration patterns on the wrist^23^. However, vibrotactile approaches have drawbacks, such as the need for relatively large coverage of different body parts, which limits spatial resolution and can result in bulky devices^22,24^. Additionally, mechanical actuators have inherent limitations in terms of rise and fall times when transitioning between amplitudes, which directly impacts the temporal resolution of vibrotactile stimulation. In contrast, electrical nerve stimulation offers much higher temporal resolution due to the rapid nature of electrical signal propagation and the absence of mechanical inertia.

Here, we present a sensory substitution paradigm implemented on a spinal computer–brain interface (SCBI). This approach leverages existing medical implants for spinal cord stimulation (SCS), which is an established therapy for chronic pain management^25–27^. However, the potential applications of SCS extend beyond pain management and offer novel therapeutic avenues for computer–brain interfacing (CBI) and sensory substitution. Utilizing implanted electrodes for CBI offers several benefits over surface-based vibrotactile methods. One of the key advantages of an SCS-based interface is its non-specific location requirement, which allows it to function at any point along the dorsal column. Moreover, the interface surpasses external wearable devices in terms of anatomical footprint and bitrate. Importantly, implantable systems can be unobtrusive, enhancing patient acceptance^28^. Recent advancements have demonstrated the efficacy of SCS devices in conveying sensory information, such as restoring sensations from a missing limb in lower-limb amputees through closed-loop stimulation^29^. Our own recent work has shown promising results in adapting SCS implants into SCBIs capable of delivering diverse sensory cues^30^. By translating visual, auditory, or tactile information into spatiotemporal electrical stimulation of the spinal cord, the SCBI induces tingling or buzzing sensations (paresthesia patterns) on the body, enabling the recipient to interpret this input in novel ways.

**Fig. 1:**
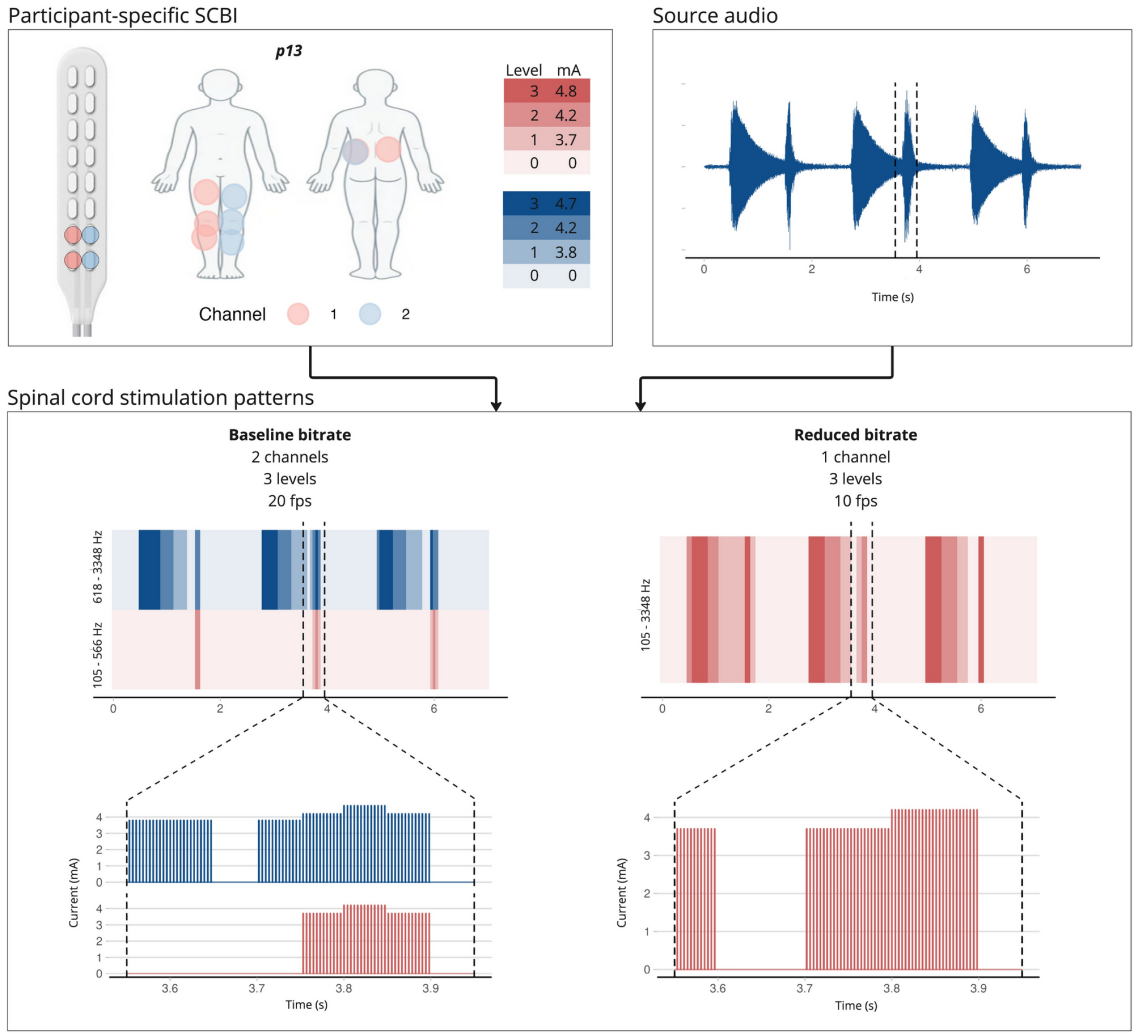
Conversion of acoustic signals into participant-specific spinal stimulation patterns. This example illustrates how an audio waveform is converted into spinal stimulation patterns, using a specific SCBI configuration for two different bitrate conditions. The acoustic signal is first divided into multiple band-pass filtered sub-signals, with each sub-signal assigned to a perceptual channel in the spinal interface. The number of frequency bands corresponds to the participant’s available perceptual channels. The resulting stimulation patterns are tailored to the individual’s specific configuration, as determined during the calibration process. Image created with ggplot2 v3.5.1 (https://cran.r-project.org/web/packages/ggplot2/) and Miro v0.8.72 (https://miro.com/). Body map background ©[sudowoodo] / Adobe Stock (Asset #503314253).

The present study investigates the utility of SCBI as a sensory substitution device for hearing. In this approach, we translate acoustic stimuli into spinal cord stimulation patterns that recipients can learn to interpret as sounds (Fig. 1). The rationale behind our method lies in the vast capacity of the somatosensory system, which processes tactile information, to encode complex sensory experiences. We hypothesize that spinal cord stimuli can evoke percepts with sufficient fidelity for reliable recognition and association with the original sounds by the recipient. To test this, we performed two experiments in a total of thirteen patients from a neurosurgery clinic, who had elected to be implanted with an SCS system for chronic pain management. We conducted the experiments during the SCS trial phase, the testing period preceding the permanent implantation of a spinal cord stimulator, during which the leads are externalized. We converted a variety of everyday sounds (e.g., ringing phones, vehicle engines, musical instruments) into personalized, time-varying stimulation inputs to the spinal cord^31,32^. These inputs were tailored based on the interface’s settings, which were determined through individual calibration, including the number of channels, intensity levels, and stimulation update rates. With calibration, we aimed to establish at least two perceptual channels, each with three or more intensity levels. We hypothesized that this approach would provide sufficient fidelity of converted sounds as spinal cord input to allow for recognition. We then first assessed the participants’ ability to recognize and identify sounds based solely on the resulting spinal stimulation patterns. In a second experiment we further investigated the role of the stimulation bitrate in the ability to discriminate between different spinal inputs.

## Results

**Fig. 2:**
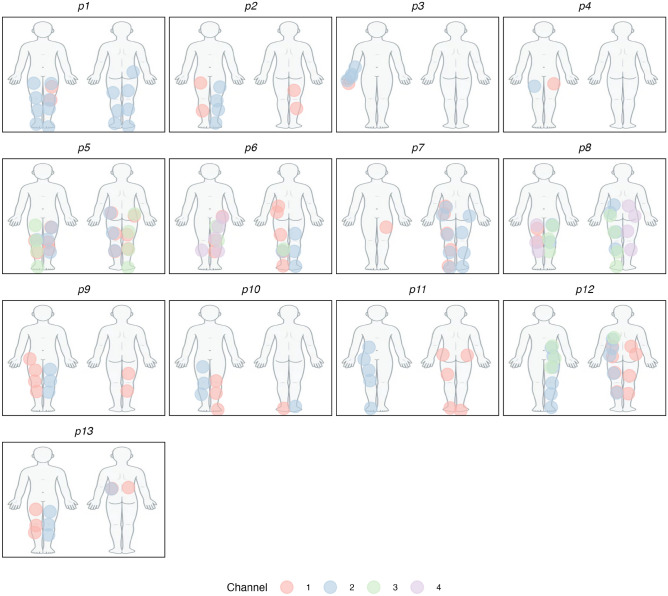
Body maps showing the locations of reported paresthesias for each of the participants. Image created with ggplot2 v3.5.1 (https://cran.r-project.org/web/packages/ggplot2/). Body map background ©[sudowoodo]/Adobe Stock (Asset #503314253).

Testing for most participants took place over several days and involved multiple sessions. In consecutive sessions, the perceptual channel settings from the initial session were verified and adjusted as needed. The body locations corresponding to the sensations of the perceptual channels are shown in Fig. 2. Participants occasionally described the sensations as an unnatural tingling, though still pleasant. However, these subjective descriptions were not systematically studied but were simply observed by the experimenter. A total of seven reports of discomfort were recorded across three participants, all occurring during the calibration phases. These accounted for 0.4% of the total calibration trials.

At least two perceptual channels were established for all participants, although not all channels were necessarily used in every experiment. Consequently, various configurations were employed across different sessions, achieving maximum bitrates between 47 and 126 bits per second, as detailed in Table 1. This variability in bitrate allowed us to assess its impact on accuracy across participants. For participants p9 to p12, who participated in experiment 2, bitrates were deliberately reduced in half of their sessions.

**Fig. 3:**
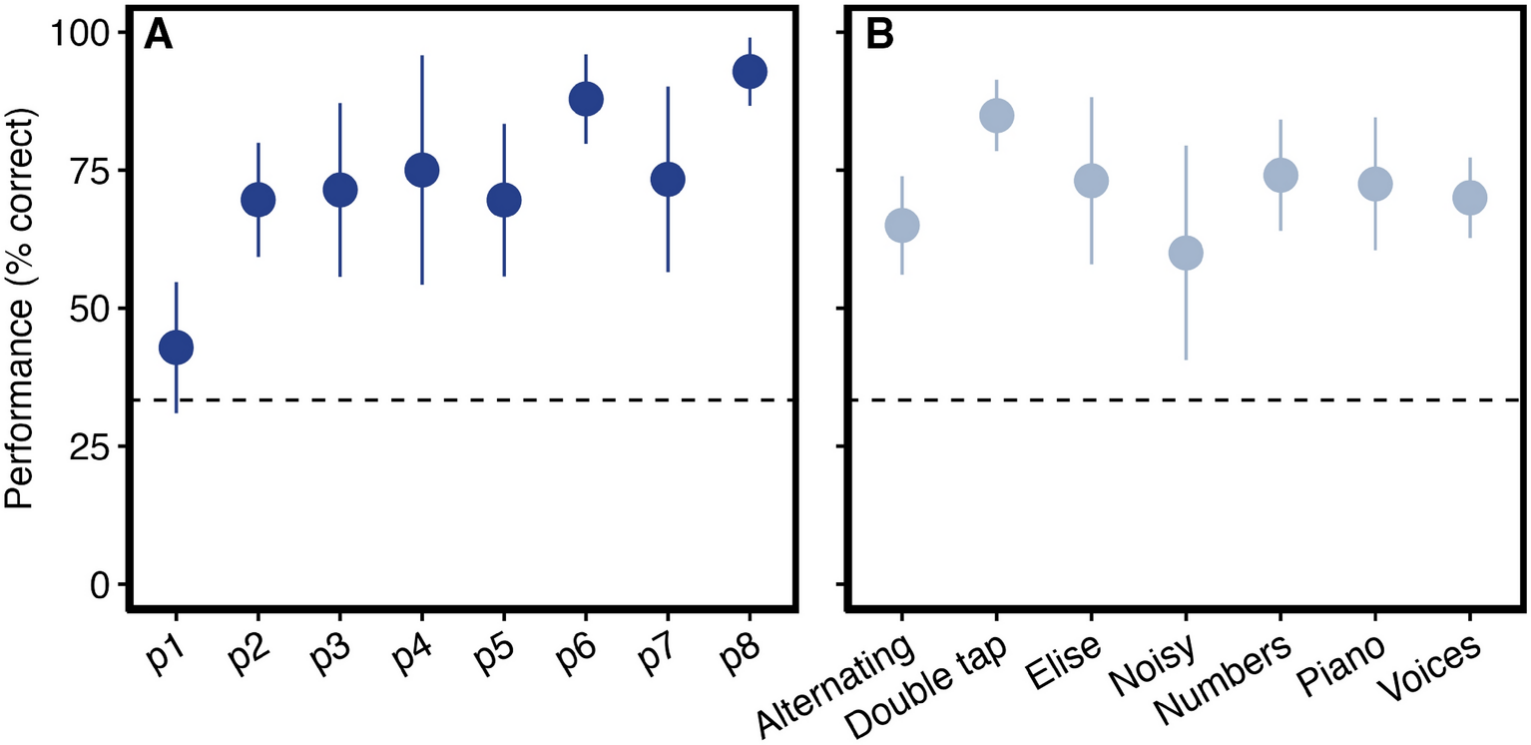
Performance in experiment 1. (**A**) per participant (**B**) per sound category. The dashed line indicates chance level.

In the first experiment, participants were tasked with recognizing and identifying sounds based solely on spinal stimulation patterns. On average, the individual total training time lasted $4.0 \pm 1.7$ min. The participants were tested on two to five different sound categories, with 20–79 trials per participant. Performance ranged from 42.9% to 92.9% correct responses, which was significantly above the 33% chance level for seven of the eight participants (all p < 0.001), except for p1, who achieved 42.9% ($p =0.061$). Table 2 summarizes the results for all participants. Figure 3 illustrates the performance rates per participant and per sound category. The overall mean performance was $72.8\pm 15\%$, significantly higher than chance ($p = 0.004$, Wilcoxon signed rank test), with a 95% confidence interval of 60–85%. Our analysis further revealed that the variability in bitrate among participants was significantly related to recognition accuracy. A logistic linear mixed effects model indicated a significant effect of bitrate on the odds of correct stimulus identification ($W(6) = 17.7; p = 0.007$), while the effect of stimulus category was not significant ($W(6) = 2.4$). Correct responses were more likely with increasing bitrate ($\text{Spearman's } \rho = 0.22, p < 0.001$; with a 95% confidence interval of 0.13–0.31).

**Fig. 4:**
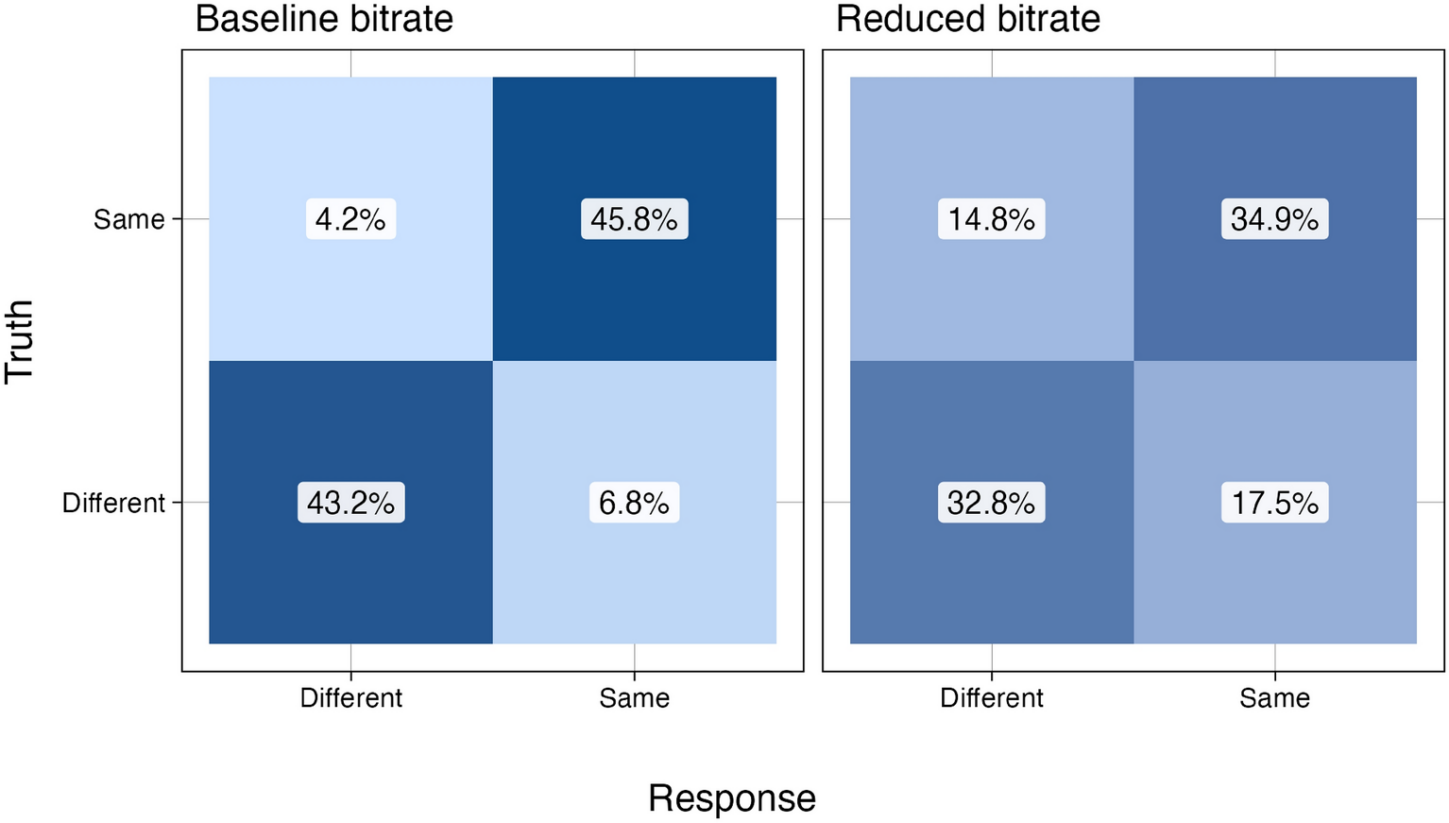
Experiment 2. Confusion matrices of the participants’ comparison of pairwise spinal sound stimuli in two different bitrate conditions.

Given the observed association between bitrate and performance, we conducted a second experiment to explore the impact of stimulation bitrate on the ability to discriminate between different spinal inputs. Participants were asked to determine whether two sequential stimuli were the same or different under two distinct bitrate conditions (baseline vs. reduced). Table 3 summarizes the individuals’ performance levels under these conditions. Mean performance at baseline bitrate was $88.3\pm 6\%$ correct responses, compared to $68.3\pm 16\%$ at reduced bitrate. The linear mixed effects analysis confirmed that correct identification of identical or different pairs depended on stimulus bitrate ($W(1) = 24.7; p < 0.001$). As shown in Fig. 4, reduced bitrate was associated with lower performance, i.e. with higher false positive and false negative rates compared to baseline. Likewise, the mean $d'$ score decreased significantly from $2.33 \pm 0.53$ at baseline to $0.98 \pm 0.90$ at reduced bitrate ($t(4) = -3.4; p = 0.014$), indicating decreased sensitivity to differences at reduced bitrates.

These findings demonstrate that SCBI can effectively convey sound information through spinal cord stimulation, with performance closely tied to stimulation bitrate, suggesting the potential of this approach as a novel sensory substitution method for hearing impairment.

## Discussion

Our study explored the use of an SCBI as a sensory substitution device for hearing, translating sound into SCS patterns that recipients could interpret. The results demonstrate significant potential for SCBI to aid individuals with hearing loss by enabling sound recognition and differentiation through spinal stimulation and could be a critical first step on a path to develop a complete prosthetic system. Recipients successfully identified sounds at rates significantly above chance, with performance correlated to the bitrate of the spinal input. Furthermore, recipients were better at differentiating consecutive sounds when these were provided at higher bitrates. This indicates that individuals were effectively processing the additional information available with higher bitrates, demonstrating a crucial aspect of the SCBI’s information processing capabilities. To our knowledge, this is a novel finding for SCBI. Overall, our findings suggest that SCBI can convey sound information with sufficient fidelity for practical use.

In experiment 2, we demonstrated that reducing the bitrate leads to significant information loss, underscoring that an adequate bitrate is crucial to maintain the fidelity of the sound information that is conveyed through the interface. However, bitrates achieved in our study are substantially lower than those typically used with CIs, which operate in the range of kilobits per second^33^. Despite this, our participants were able to achieve sound recognition rates significantly above chance levels, indicating that with the present limited level of complexity, the SCBI can provide meaningful sound information. The data from experiment 2 indicate that lowering the bitrate led to a decline in participants’ performance. However, the inverse may not necessarily hold true. With the average performance at the baseline bitrate already at 88%, there is likely a ceiling beyond which further increases in bitrate will not result in significant improvements, especially for relatively simple tasks like those used in this experiment. Nevertheless, more demanding situations or complex sounds may require higher information throughput rates. Further improvements in bitrate and signal fidelity could enhance SCBI’s performance, which could be a critical step on a path to develop complete prosthetic system.

While SCS has proven effective in chronic pain management, this research expands upon our earlier demonstration of the feasibility of a general-purpose computer–brain interface paradigm using established medical SCS platforms^30^, revealing broader and potentially transformative applications for SCBI. By demonstrating its suitability for sensory substitution, SCBI opens new doors for treating various sensory deficits, potentially benefiting a wider patient population. If successful, SCBI could revolutionize the way we address hearing loss, offering an alternative method to restore sound perception and significantly enhancing the quality of life for individuals who are not candidates for CIs or do not achieve sufficient benefit from them.

**Fig. 5:**
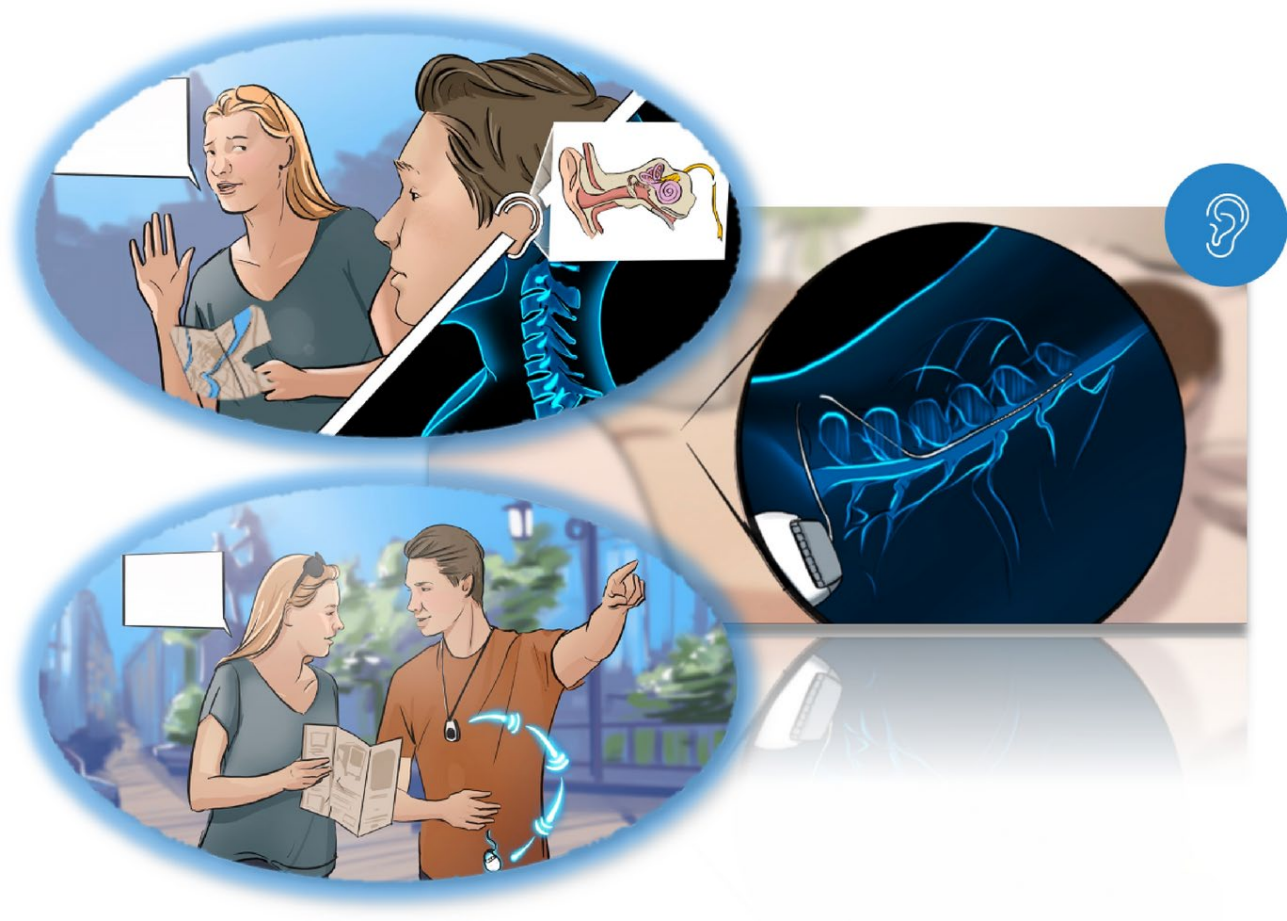
Artist impression of potential real-world applications of the SCBI hearing aid. The SCBI provides the recipient with sound related information or cues through stimulation of afferent pathways of the spinal cord. People with sensorineural hearing loss due to inner ear conditions, such as neurofibromatosis type 2 or vestibular schwannoma, may benefit from this intervention.

Importantly, the SCBI surpasses external wearable sensory substitution devices in terms of spatial footprint^24^ and achievable bitrate^22^, offering a more compact and efficient solution. An SCBI system for hearing would focus on stimulating the large A$\beta$ axons in the dorsal columns or in the dorsal roots of the spinal cord^34,35^. A commercial SCBI system for hearing substitution would share some similarities with a CI. Instead of targeting the auditory nerve, it would address the spinal cord, as illustrated in Fig. 5. An external device with a microphone would capture environmental sounds, which a digital signal processor (DSP) would process into specific stimulation patterns tailored to the individual’s spinal cord interface. These processed signals would then be transmitted wirelessly to the implanted stimulator, delivering electrical pulses to the spinal cord via implanted electrodes. Real-time processing and feedback are crucial for providing a seamless user experience. We acknowledge that developing wireless communication between an external microphone and an implanted stimulator is a non-trivial engineering challenge, particularly in ensuring reliable signal transmission through biological tissue, minimizing implant power consumption, and achieving sufficient bandwidth and low latency for real-time auditory processing. An initial 2–3 week trial phase is common practice in SCS pain therapy^36,37^. Similarly, an SCBI trial period could allow candidates to experience the benefits before deciding on permanent implantation, ensuring informed decision-making and personalized care.

Combining SCBI with existing auditory prostheses could offer a more comprehensive auditory experience. SCBI can complement residual auditory function and enhance perception alongside CIs by providing low-frequency sound information to complement high-frequency auditory signals. This could benefit adults with profound high-frequency hearing loss and moderate to severe low-frequency hearing loss^5^. Many CI recipients receive insufficient low-frequency sound information, making SCBI a valuable addition^38^. For CI users who struggle with specific sound recognition or in noisy environments, SCBI could provide supplementary sound-related input. Several studies have demonstrated that speech-in-noise understanding in noisy environments can be enhanced when auditory speech signals are complemented with vibrotactile skin stimulation^39–45^, sometimes requiring minimal or no training at all^46,47^. Therefore, we believe that SCBI may provide synergistic benefits when used alongside CIs. For patients who are not candidates for CIs (or ABI), SCBI offers an alternative pathway for sound information. Our approach may be particularly appropriate for individuals with neurofibromatosis type 2 (NF2) and vestibular schwannomas (VS). Both NF2 and VS are often associated with hearing impairments, primarily due to damage to the auditory nerve. This nerve damage makes these patients poor candidates for CI, which require a functional auditory nerve to be effective^48^. SCBI bypasses the auditory nerve and directly stimulates the spinal cord, offers a promising alternative for these patients by providing a novel pathway for sound perception. Given that these diseases result in hearing impairments that are not amenable to traditional cochlear implant treatment, SCBI may offer a viable solution, filling an unmet clinical need for those with nerve-damage-induced hearing loss^49^. Finally, although a direct comparison of risk profiles is lacking, the minimally invasive nature of the envisioned SCBI surgery suggests it could offer a safer alternative to ABI. The low adoption rate of ABI is likely partly due to its high costs, surgical complexity, significant risks, and complications. Noij et al.^50^ reported that major complications occurred in 20.8% of pediatric ABI recipients. In contrast, SCS procedures and their technology are well-established, widely available, and regarded as relatively safe, as evidenced by their extensive use in chronic pain management^51,52^. Therefore, SCBI could lead to greater adoption over ABIs by providing an effective solution with reduced invasiveness and improved safety.

There are several important limitations of our study that must be considered. Firstly, the sample size was relatively small, potentially limiting the generalizability of the findings to the broader population or specific groups of individuals with hearing loss. This limitation arose from (necessarily) recruiting participants exclusively from patients who underwent SCS implantation for pain management, all of whom lacked pre-existing hearing impairments. Additionally, our results were obtained with implant locations chosen for optimal pain treatment, using off-the-shelf electrodes that were not specifically optimized for this purpose. Consequently, we likely did not reach the maximum potential for information transmission. Furthermore, the study utilized a limited set of sound stimuli. While participants in experiment 1 encountered multiple stimuli from various categories, the experimental design restricted the mixing of stimuli within each training and test block to only three items. Additionally, the assignment of sound categories was not systematically controlled. Although each participant was tested with at least two different categories to cover a broad range of sounds, a more systematic approach would improve methodological rigor. Training and exposure durations were relatively brief, with experiment sessions that lasted just several minutes. Future research should explore sound identification with different and larger sets of stimuli and investigate extended training and exposure duration. Moreover, there is a need to investigate the potential for achieving open-set sound recognition abilities, for example by employing speech-in-noise tests^53^. Previous research has shown that individuals with chronic neuropathic pain often exhibit altered sensory thresholds and heightened sensitivity due to central sensitization, which can affect pain processing even in non-injured areas^54,55^. In our study, these altered pain processing pathways may have influenced the perception of paresthetic sensations, potentially limiting the generalizability of our results to pain-free individuals. Despite the stimulation primarily overlapping with the painful areas targeted for clinical SCS, participants generally reported the induced paresthesias as pleasant, and discomfort was rarely reported during stimulation. Further research on SCS-induced paresthesias in non-pain populations is necessary to provide a more comprehensive understanding of the SCBI.

Moreover, we believe that conscious perception of paresthesias is a crucial component of the SCBI paradigm. While participants in our experiments generally found the sensations to be unnatural but not unpleasant, it remains uncertain whether this will hold true in real-world, everyday use. A key difference between conventional SCS for pain management and SCBI is that conventional SCS delivers continuous, unchanging stimulation, whereas SCBI provides highly variable stimulation based on environmental sound inputs. This dynamic nature may influence how users perceive or tolerate the sensations. We speculate that the functional utility of SCBI-induced sensations may influence how recipients perceive and interpret them. Supporting this, studies have shown that initial electro- or vibrotactile stimulation can be distracting due to its novelty, but training and familiarization improve its integration with auditory information^41,56^. Thus, conscious perception of sensory feedback can, through training, become a useful and non-distracting input that enhances the user’s ability to interpret sensory information. A similar learning effect could be expected in SCBI applications, though this has yet to be tested.

In conclusion, our study demonstrates that SCBI can effectively convert sound into interpretable spinal cord stimulation patterns, offering a novel approach to sensory substitution for individuals with hearing loss. While current bitrates are lower than those of CIs, the performance levels achieved are encouraging. Using non-optimized electrodes and pain-treatment implant locations likely limited our results, suggesting potential improvement with optimized designs and placements. In addition to accuracy, response speed is crucial for practical applications. Therefore, future studies will incorporate reaction time measurements to assess both the speed and accuracy of participant responses. Reaction time is a well-established biomarker for cognitive functions such as attention, processing speed, and decision-making, making it an important factor in evaluating the SCBI’s overall functionality. Enhancement of the SCBI’s bitrate and functionality could make it a viable complement or alternative to existing auditory prostheses. The personalized nature of spinal interfaces, potential integration with residual auditory function, and relatively low risk profile highlight the promise of SCBI as part of a diverse range of treatment options for hearing loss. Future studies should focus on increasing bitrates, improving stimulation fidelity, and assessing long-term durability, reliability, and training effects. Integrating SCBI with other hearing aids, such as CIs, could provide a comprehensive approach to auditory rehabilitation.

## Methods

### Participants

We recruited patients from the Institute for Neurosurgery and Neurointervention of Semmelweis University (formerly named National Institute of Mental Health, Neurology and Neurosurgery / Országos Mentális, Ideggyógyászati és Idegsebészeti Intézet), who were electing to undergo SCS procedures for treatment of chronic neuropathic pain. We included thirteen patients in total. All participants provided written informed consent after the nature and possible consequences of participation in the study was explained, and prior to the experimental procedures, which took place between 9 February 2023 and 19 June 2024. The basic demographic characteristics and relevant clinical background of the participants are listed in Table 4. Seven participants were of female sex, the other six were male. Their age at the first experiment day ranged from 28 to 72 years (median 54 years).

We performed our experiments with externalised leads, during the SCS trial phase. For twelve participants, the arrays were placed surgically into the epidural space (lead model Artisan; Boston Scientific, Marlborough, MA, USA). For one participant, the array (lead model Linear ST; Boston Scientific) was placed percutaneously. We conducted the experiments in accordance with local guidelines and regulations and in accordance with the Declaration of Helsinki. The NIMHNN institutional review board, national ethics council (TUKEB) and Hungarian Ministry of Health (OGYÉI) approved the study (agreement number OGYÉI/23818/2019).

### Experimental set-up

Experiments took place in a laboratory room in the clinic. The participant sat on a chair, behind a regular office desk. At about 60–80 cm from the participant, there was a PC monitor to provide feedback to the participant during the experiment. We played auditory stimuli through a PC speaker at about 50–80 cm away from the participant. We applied spinal cord stimulation using an external programmable neurostimulator (CereStim; BlackRock, Salt Lake City, UT, USA). We connected the neurostimulator to the SCS lead through a disposable sterile cable. We used custom software written in Matlab (MathWorks, Natick, MA) to send instructions to the CereStim neurostimulator interface using the manufacturer’s software development kit. We used a central control computer running Microsoft Windows and custom Matlab software, including the Psychophysics toolbox^57^, to coordinate and log the delivery of stimuli and to register experiment events and responses.

### Spinal cord stimulation

We applied stimulation using either a multipolar configuration, meaning that both the cathode(s) and anode(s) were located on the SCS electrode array, or a monopolar configuration, in which a self-adhesive electrode pad on the participant’s skin was used as the counter-electrode. The stimulation parameter range was restricted to the established safety ranges, and not beyond settings allowed in implantable spinal cord neurostimulators. All stimulation pulses were bi-phasic. Active charge balancing was achieved by providing an opposite-polarity recovery pulse directly after each primary stimulation pulse. The median and range of the stimulation parameters were: pulse frequency 255/[120–450] Hz, pulse width 150/[70–450] $\upmu \hbox {s}$, single contact pulse amplitude 2.3/[0.5–6.0] mA, total amplitude 3.8/[1.5–9.0] mA.

For the SCBI we used stimulation settings that evoked reproducible sensations in distinguishable dermatome regions. We term such reproducible stimulus-sensation relations the ’perceptual channels’ of the spinal interface. The procedure to establish perceptual channels is described in detail in^30^. Here we followed this procedure with the aim of obtaining in each participant at least two perceptual channels with each having at least three intensity levels. In some cases, we extended the calibration to yield more perceptual channels and/or levels.

During this calibration, participants were asked to verbally report their experience after each short stimulation, including whether or not they felt a paresthesia, the relative intensity of the sensation, and the body regions affected. Stimulation-induced discomfort was also recorded, if occurring. To ensure participant comfort and practicality, these verbal reports were provided after each trial and logged by the experimenter using an interactive dermatome map on the computer system. Additional perceptual qualities of the stimulation-evoked sensations were not systematically recorded.

### Conversion of sound to spinal cord input

**Fig. 6:**
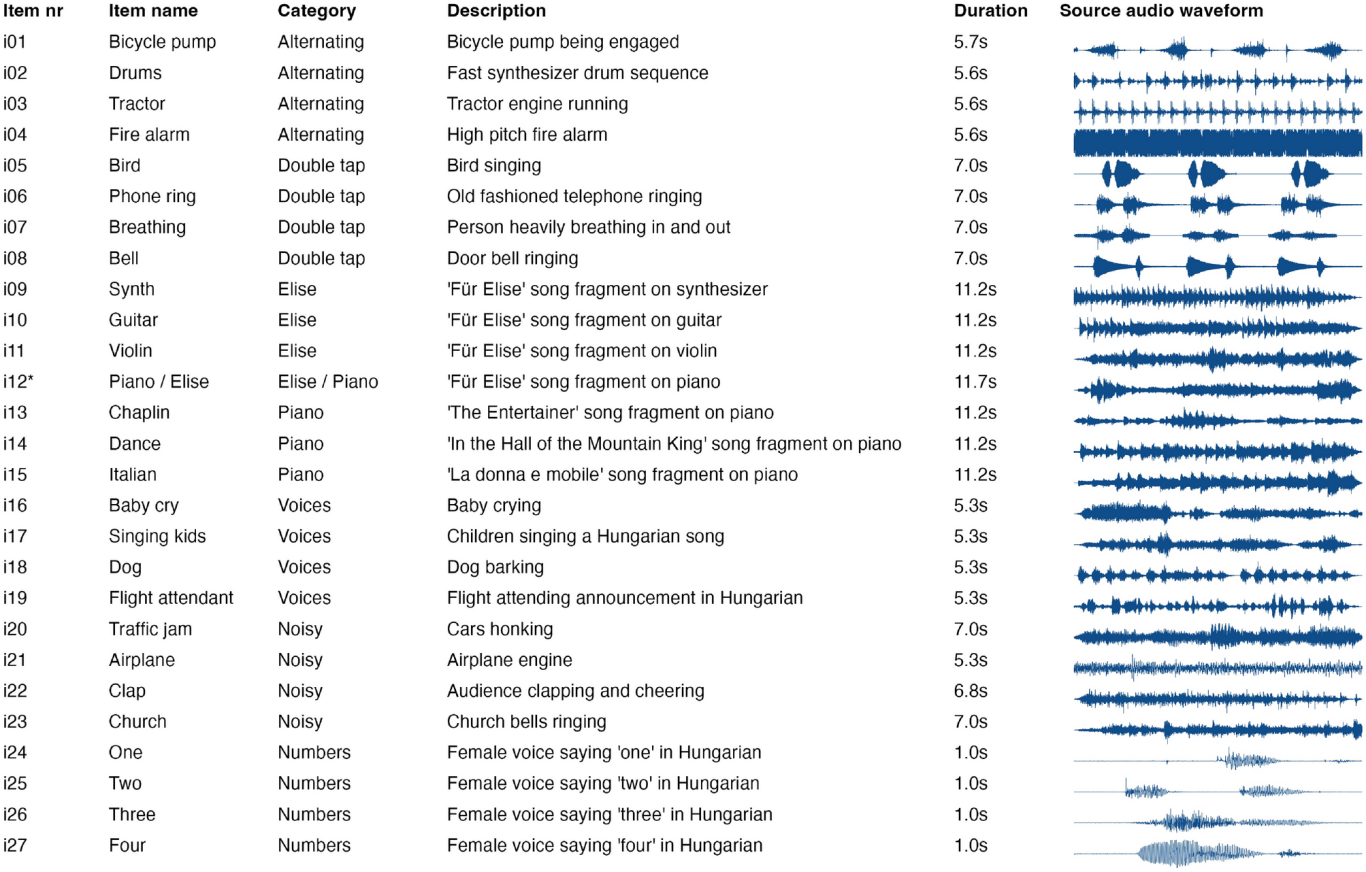
The collection of sound sample items used for the experiments, grouped by category. In experiment 1, each participant was trained and tested on subset of this collection. Item i12^*^ was used in both the ‘Elise’ and ‘Piano’ categories; however, no participants were tested on both categories. In experiment 2, we used the items from the “Double tap” category.

After the perceptual channels were established and validated, each acoustic wave file (see Fig. 6) was transformed into time-varying multi-channel electrical stimulation patterns that could be transmitted through the spinal interface. Figure 1 illustrates this process with an example. The sound transformation was done with continuous interleaved sampling (CIS), which is a method from the cochlear implant domain^31,32^. With this method, the acoustic signal is divided into multiple band-pass filtered subsignals. Each band-pass filtered subsignal is then converted into electrical impulses that can be sent through the spinal interface. In CIS, the channels are activated in an interleaved manner, meaning that electrodes from different perceptual channels are stimulated in succession rather than simultaneously. This helps to minimize interactions between perceptual channels, and prevents unintended summation of stimulation pulses across electrodes from different perceptual channels. We assigned each of the subsignals to a different perceptual channel. The number of frequency bands we used to split the acoustic sample was equal to the number of perceptual channels the spinal interface for a given individual provided. The electrical stimulation patterns that resulted from the transformation of the acoustic source files were participant-specific, since this depends on the perceptual channel configuration, intensity levels, and stimulation update rates that are determined in the calibration phase, and was specific for each individual and/or experiment session. The electrical stimulation pulses stimulate the large A$\beta$ axons in the dorsal columns or in the dorsal roots of the spinal cord^34^, thereby causing a temporal variation in paresthesia sensations across different body regions. The participants were trained and tested to identify and discriminate the different temporal patterns of paresthetic sensations.

### Procedure experiment 1

In this experiment, participants were tested on their ability to identify sounds that were converted into electrical stimulation patterns delivered through the spinal cord interface. As shown in Table 1, several bitrates were used both within and across participants in different experiment sessions. Bitrates were based on the number of perceptual channels established for each participant and the specific configurations employed in each session. The experiment was conducted in blocks, each using 3 different sound stimuli from a single category (see Fig. 6), presented in a random order. During each trial, a single sound item was presented, and the participant was asked to identify it. The participant was presented with two or more blocks of items from different sound categories, with at least 10 trials per category. While the selection was not systematically randomized and was left to the experimenter’s discretion, we ensured that each participant was tested with at least two different categories, varying these among participants to cover a broad range of sounds within our limited sample size. Prior to the test phase, participants received general instructions about the nature of the task and what constituted a correct or incorrect answer. In each block, all stimulus items of the respective category were first presented in their canonical form (i.e., as sound). Simultaneously with each sound stimulus, a visual icon was presented on the computer screen to facilitate identification. No spinal cord stimulation was applied during this familiarization phase. This phase continued until the participant indicated that she/he was familiar with each of the sound items and could identify them. In the subsequent training phase, each item was presented as a combination of the canonical sound, the corresponding visual icon, and its associated electrical spinal cord stimulation pattern. Items were repeated until the participant indicated they could recognize each of the three spinal cord stimulation patterns. Finally, in the test phase, participants were required to identify the items solely based on their associated spinal cord stimulation patterns. Participants responded verbally and their response was rated for correctness.

### Procedure experiment 2

In this experiment, we aimed to investigate whether the bitrate of the spinal cord input affected participants’ ability to distinguish between different stimulation patterns. We hypothesized that reducing the bitrate would decrease the ability to distinguish stimuli. We used stimuli from the “Double Tap” category (see Fig. 6). Each participant performed the experiment with the spinal interface configured at two different bitrates: the baseline bitrate and a reduced bitrate. The experiment used a blocked design, and to control for potential order effects, the sequence of bitrate conditions was counterbalanced. The baseline bitrate was determined during the calibration phase. For the baseline bitrate we typically used all perceptual channels and levels available in the respective setting, with a stimulation update rate of 20 frames per second. The reduced bitrate was set to half of the baseline, or less. In each trial, two sequential stimuli were presented, at the same bitrate. Participants had to compare the two stimulation patterns and report whether they were identical or different. Each stimulus and stimulus pair was used equally often, and the ratio of same vs. different pairs was 50-50%. Before the test phase, participants underwent a familiarization procedure, for both bitrates, similar to that described for experiment 1. During the test phase, participants responded verbally, with only two possible response options: “Same” or “Different” (i.e., a forced-choice task), and their response was rated for correctness.

### Statistical analyses

We report descriptive statistics as averages followed by the associated standard deviation, unless stated otherwise. The participants’ performance was quantified as the percentage of trials in which the stimulus was correctly identified. For experiment 2, we also report the $d'$ sensitivity index, which reflects the individual’s ability to discriminate the stimulus pairs, while taking response bias (i.e. tendency to favor one response option over another) into account^58^. A $d'$ of zero indicates an inability to distinguish, whereas larger positive values indicate better discrimination ability. Negative values can occasionally occur, for instance, due to response confusion.

To statistically evaluate individual participant performance in experiment 1, we used a one-sided binomial test to assess the null hypothesis that the percentage of correct responses does not exceed the chance level of 33.3% (1/3). We used a one-sided Wilcoxon signed-rank test to evaluate whether the group-wise performance exceeded chance level. To assess whether the bitrate was associated with the odds of a correct response on a given trial, while accounting for variance introduced by other variables such as participant identity and stimulus item, we employed generalized linear mixed model logistic regression analyses. For correlations, we used Spearman’s $\rho$ coefficient, which reflects the rank order correlation between variables. We used a one-tailed paired Student’s t-test to evaluate the effect of bitrate reduction on the $d'$ scores. For all statistical tests, the significance level ($\alpha$) was set at 0.05. Error bars in figures reflect the 95% confidence interval of the mean. We performed all statistical analyses with R version 4.4.

**Table 1:** Summary of spinal cord interface settings used in the different experiment sessions.

Participant	Session	Channels	Total nr of intensity levels	Bits per frame	Framerate	Bitrate
p1	1, 2	2	6	3.2	20 fps	63 bps
p1	2	2	10	4.6	20 fps	92 bps
p2	1, 2, 4	2	6	3.2	20 fps	63 bps
p2	3	2	7	3.6	20 fps	71 bps
p3	1	2	6	3.2	15 fps	47 bps
p4	1	2	12	5.2	20 fps	103 bps
p5	1, 2	3	9	4.8	20 fps	95 bps
p6	1, 2, 3	3	9	4.8	20 fps	95 bps
p7	1	2	6	3.2	20 fps	63 bps
p8	1, 2	4	12	6.3	20 fps	126 bps
p9	1, 2	1	3	1.6	10 fps	15 bps
p9	1, 2	2	6	3.2	20 fps	63 bps
p10	1	2	6	3.2	20 fps	63 bps
p10	2	2	6	3.2	10 fps	31 bps
p11	1	1	3	1.6	10 fps	15 bps
p11	2	2	6	3.2	20 fps	63 bps
p12	1	1	3	1.6	5 fps	7 bps
p12	1, 2	2	6	3.2	20 fps	63 bps
p12	2	1	3	1.6	10 fps	15 bps
p13	1, 2	1	3	1.6	10 fps	15 bps
p13	1, 2	2	6	3.2	10 fps	31 bps
p13	1, 2	1	3	1.6	20 fps	31 bps
p13	1, 2	2	6	3.2	20 fps	63 bps

**Table 2:** Results of experiment 1.

Participant	Training time (mins)	Categories tested	Nr of test trials	Percentage correct
p1	2.3	Alternating, numbers, voices	70	42.9% (.)
p2	2.3	Alternating, noisy, numbers, piano, voices	79	69.6% (***)
p3	3.5	Alternating, double tap, elise	35	71.4% (***)
p4	1.8	Noisy, voices	20	75.0% (***)
p5	5.0	Alternating, double tap, elise, voices	46	69.6% (***)
p6	5.6	Double tap, numbers, piano, voices	66	87.9% (***)
p7	6.3	Alternating, double tap, piano	30	73.3% (***)
p8	5.2	Alternating, double tap, numbers, piano, voices	70	92.9% (***)

(.) $$0.05< p < 0.1$$; (***) $$p<.001$$.

**Table 3:** Experiment 2.

Participant	Bitrate	Nr of pairs	Percentage correct	$d'$
p9	Baseline	48	87.5%	2.20
p9	Reduced	48	72.9%	1.18
p10	Baseline	24	79.2%	1.48
p10	Reduced	21	66.7%	0.79
p11	Baseline	24	91.7%	2.64
p11	Reduced	24	75.0%	1.27
p12	Baseline	48	89.6%	2.47
p12	Reduced	48	41.7%	-0.41
p13	Baseline	48	93.8%	2.84
p13	Reduced	48	85.4%	2.05

Performance per participant for the two different bitrates.

**Table 4:** Patient demography and implantation information.

Experiment	Participant	Sex	Age	Lead type	Lead vertebral level	Placement
1	p1	Female	55	Artisan	T8–T9	Surgical
1	p2	Female	72	Artisan	T8–T9/10	Surgical
1	p3	Male	48	Linear ST	C4–C4	Percutaneous
1	p4	Male	56	Artisan	T8/9–T10	Surgical
1	p5	Female	49	Artisan	T8–T9/10	Surgical
1	p6	Female	63	Artisan	T9–T10	Surgical
1	p7	Female	42	Artisan	T9–T10	Surgical
1	p8	Female	53	Artisan	T8/9–T10	Surgical
2	p9	Male	54	Artisan	T8–T9	Surgical
2	p10	Male	62	Artisan	T9–T10	Surgical
2	p11	Female	28	Artisan	T8–T9	Surgical
2	p12	Male	48	Artisan	T7–T8	Surgical
2	p13	Male	54	Artisan	T7–T8	Surgical

## Data Availability

The datasets generated during and/or analysed during the current study are available from the corresponding author on reasonable request.
